# Multitechnique Approach to Trace Characterization of High Purity Materials: Gallium

**DOI:** 10.6028/jres.093.093

**Published:** 1988-06-01

**Authors:** S. Gangadharan, S. Natarajan, J. Arunachalam, S. Jaikumar, S. V. Burangey

**Affiliations:** Ultratrace and Nuclear Methods Section, Analytical Chemistry Division, Bhabha Atomic Research Centre, Trombay, Bombay 400 085 India

## 1. Introduction

Compositional characteristics complement the structural aspects and in combination with certain correlated property measurements, provide complete characterization of materials. While characteristics completely govern the properties, the properties do not give a unique or complete account of the characteristics. The ultimate constraint in the characterization of ultrapure materials is the ability to maintain the integrity of the sample throughout the analytical measurement process. Developments in clean-room technology, coupled with the advances in plastics industry, have helped to push down the (environment and reagent) blank levels which has led to increased reliability in the quantitative measurement of impurities at ppb and sub-ppb levels of concentration. The stringent requirement of purity of materials for high technology demand compositional characterization for several (metallic) impurities which necessitates the use of diverse techniques like GFAAS, ICP/AES, activation analysis and electrochemical techniques, the choice of a given technique being guided by the sensitivity and selectivity it offers towards the characterization. To ensure good precision for measurements at ultratrace levels, the analytical methodologies should ensure minimum process blanks and afford (simultaneous) analysis of several critical elements related to (i) the manufacturing process, (ii) chemical similarity with reference to the matrix, and (iii) in the case of semiconductor materials, the donor, acceptor and neutral impurities.

### 1.1 The Facility

A (prototype) ultratrace analytical facility has been set up using indigenously available technology, to establish the attainable levels of cleanliness. The laboratory, designed to be at different levels of cleanliness, is better than class 100 at the work surface. The cleanliness with respect to (metallic) impurities has been monitored through the analysis of the air samples at several locations in addition to particulate counting. Distilled water, passed through a mixed bed ion exchange resin, is being routinely used. The levels of some of the impurities in that water, which reflect the cleanliness of the laboratory environment is as follows:

**Table:** 

Element	Cd	Cu	Fe	Pb	Zn
Level ng/L	3	10	60	10	100

These levels have been maintained over several months of operation.

## 2. Approaches: Experimental

The multitechnique approach to trace characterization of gallium metal has been adopted. The philosophy behind the approach has been (i) to ensure cross-validation, i.e., generation of analytical results by at least two independent analytical methodologies wherever possible and obtain complementary information, (ii) to develop optimum instrumental parameters during the final measurement, (iii) to analyze different aliquots of samples to check homogeneity and sample requirement, and (iv) analyze subsamples obtained from a large volume solution to generate an intra-lab comparison on the basis of inter-technique capabilities.

Direct analysis of Ga metal using NAA, though virtually free from process blanks, is limited to elements providing good sensitivity through long lived isotopes and requires proper configuration of the sample during irradiation to minimize flux perturbation. Analysis by ICP/AES, DPASV and GFAAS requires the sample to be brought into solution. Signal suppression of about 20% for ultratrace constituents at ~100 ng/mL is noted in ICP/AES when Ga is present in ~1 ng/mL. Suppression of anodic waves as well as non-linear behavior with respect to standard addition has also been observed in DPASV. Direct analysis of Fe, Cu, Ni etc. in Ga at >1 ppm levels is possible by GFAAS but severe signal suppression for many elements is noted for trace constituents when even tens of micrograms of Ga is present.

### 2.1 APDC/MIBK Extraction

Solvent extraction of elements as their thiocarbamates has been investigated using APDC as the complexing agent and MIBK as the extractant. The optimum pH levels for complete extraction of trace constituents from citrate as well as tartrate media (which inhibit the extraction of Ga) have been investigated using GFAAS and radioactive tracers. Optimization of parameters like the quantity of Ga taken, the amount of buffer required as well as the concentration of APDC used for complexing the metals, was carried out to ensure that virtually no Ga extracts into the organic layer. The MIBK layer was directly analyzed by GFAAS. The tartrate medium provided a more consistent and effective separation in addition to reduced blanks than citrate medium. Current levels of the process ‘blanks’ for some trace elements (1-g sample basis) are given below:

**Table:** 

Element	Cd	Co	Cu	Ni	Pb
blank levels (ppb)	1	8	45	40	70

### 2.2 Preconcentration of Trace Impurities on Ga Itself through Controlled Dissolution

A 1-g Ga sample was dissolved slowly in a 3:0.15 M mixture of HCl:HNO_3_ at 95 °C for several hours until a residue of ~10 mg remained. This residue was carefully washed and weighed, dissolved in an HCl:HNO_3_ mixture and aliquots of this solution were directly analyzed by GFAAS, ICP and DPASV. Theoretically this procedure should provide the least process blank, limited by the purity of the acids used. The process ‘blanks’ (1-g equivalent) as analyzed by ICP/AES and GFAAS are given below:

**Table:** 

Technique/Element	Ag	Bi	Cu	Fe	Ni	Pb	
GFAAS	4	–	40	10	14	10	(ng/g)
ICP	–	90	40	–	–	–	

### 2.3 Preconcentration of Impurities in Aqueous Medium through Solvent Extraction of Ga in MIBK

Methyl isobutyl ketone is an excellent extractant of Ga from concentrated HCl medium. Our choice of MIBK as the extractant has been mainly due to much lower molarity of HCl required under similar operating conditions.

- 99.99% extraction of Ga (5 M) HCl, compared to di-isopropyl ether (7.6 M) and chlorex (12 M) and hence lower blanks.
- Good phase separation compared to ether.
- Presence of small amount of nitrate does not hamper Ga extraction; close to 80% recovery of Cd, Co, Cr, Cu, Mg, Mn, Zn at ~100 ng/mL level in presence of 250 mg Ga is obtained. The process ‘blanks’ (1-g sample equivalent) estimated by GFAAS and ICP are given below:

**Table:** 

Element (ppb)	A1	Ca	Cd	Co	Cu	Mg	Mn	Ni	Pb	V	Zn
Technique ICP	60	20	20	30	40	15	15	60	–	30	40
GFAAS				11			10	20	50

### 2.4 Other Approaches

Solvent extraction of Ga with ethyl-hexyl-phosphoric acid (DEHPA) affords preconcentration of more elements than MIBK and the extraction can be done at very low acidities. Currently, the purification of the reagent for suitability to work at nanogram levels is being attempted. Coprecipitation of several impurities by reductive precipitation with Pd under alkaline conditions appears very promising, but even pure PdCl_2_ contains several trace impurities that need to be removed. An ion exchange procedure has been developed to purify PdCl_2_ for nearly complete removal of several trace impurities, and efforts to minimize process blanks have been undertaken.

## 3. Results

Results on two of the samples characterized are summarized in tables 1 and 2. Current levels of process blanks (1-g basis) by various procedures described here indicate that the lab environment and analytical methodologies are capable of handling Ga samples of 99.9999 + % purity, with respect to some specific impurities. While the purity required is related to end-use, we wish to point out the levels of Hg and Zn impurities in two samples. Ga samples obtained from a single source at different times showed varying levels of Hg, which appears to be related to sampling errors. Another sample, though very pure with respect to several elements, contained about 115 ppm of Zn which may be a source-related impurity. Similarly, levels of Al, V and Pb also appear to be source-related.

## 4. Comments

The restrictive nature of the presence of gallium in suppressing analytical signals in ICP/AES, DPASV and GFAAS, requires the development of optimum conditions for sample processing and final measurement. Optimization techniques based on simplex and method of steepest ascent are currently being examined to develop an experimental design which permits (1) preconcentration of several trace impurities with virtually no Ga accompanying them, (2) identifying analysis conditions in ICP/AES and GFAAS that afford signal enhancement with reduced matrix interference with and without modifiers. These results will be communicated shortly.

## Figures and Tables

**Table 1:** Trace impurities (ppb) in gallium (sample A) (analytical techniques INAA(a), GFAAS(b), ICP/AES(c), DPASV(d))

Element	Direct	Cont. dissolution	MIBK ext.	APDC/MIBK
Ag		30(b)		
Au	76(a)	35(a). < 100(b)		
Bi		50(c)		
Cd			4.2	7(b)
Co	7(a)		17(b)	12(b)
Cr	11(a)			
Cu		190(b), 180(c)	280(b)?	190(b)
In	2.8(a)ppm			
Mn			< 24(b), 25(c)?	
Ni		725(b), 590(c)	740(b)	820(b)
Pb		25(b), 60(c)	20(b)	40(b)
		60(d)?		
Pd		<60(b)		
Sb	<3(a)	15(a)?		
Zn	300(a)			

**Table 2:** Trace impurities (ppb) in gallium (sample B) (analytical techniques INAA(a), GFAAS(b). ICP/AES(c), DPASV(d))

Element	Direct	Corn. dissolution	MIBK ext.	APDC/MIBK
Au	0.4(a)			
Bi		<50(c)		
Cd			2.4	5.5(b)
Co	5(a)		<11(b)	<8(b)
Cr	7(a)			
Cu		85(b), 50(c)		43(b)
In	84(a)			
Mn			16(b)	
Ni		< 14(b), < 100(c)		<40(b)
Pb		26(b), 60(c)	<10(b)	<100(b)
Sb	15(a)	26(a)?		
Zn	106 ppm(a)		114 ppm(a), 100 ppm(c)	

